# Epidemiology and Risk Factors for Viral Infections in Pediatric Liver Transplant Recipients and Impact on Outcome

**DOI:** 10.3390/v15051059

**Published:** 2023-04-26

**Authors:** Hala Abdullatif, Anil Dhawan, Anita Verma

**Affiliations:** Pediatric Liver Unit, King’s College Hospital, London SE5 9RS, UK

**Keywords:** liver transplantation, CMV, EBV, non-EBV-CMV viral infections

## Abstract

Infections after liver transplantation (LT) are risk factors for morbidity and mortality. Infections, especially of viral etiologies, still have an impact on the graft function and overall outcome. The aim was to review the epidemiology and risk factors of EBV, CMV and non-EBV non-CMV viral infections and their impacts on outcomes after LT. Demographic, clinical, and laboratory data were retrieved from patients’ electronic databases. Over 2 years, 96 patients were transplanted at the Pediatric Liver Centre at Kings College Hospital. The majority of the infections were of viral origin; 73 (76%) patients. The incidence of EBV viremia was 60.4%, CMV infection 35.4%, and other viruses 30%. Older donor age, auxiliary graft, and bacterial infections were risk factors for EBV infection. Younger recipient age, D+R− CMV IgG, and left lateral segment graft were risk factors for CMV infection. More than 70% of patients with non-EBV and CMV viral infections stayed positive post-LT but did not contribute to increased complications. Despite the high prevalence of viral infections, EBV, CMV, and non-EBV non-CMV viral infections were not associated with rejection, morbidity, or mortality. Although some of the risk factors for viral infections are unavoidable, identifying the characteristics and risk pattern will help improve the care for pediatric LT recipients.

## 1. Introduction

Liver transplantation (LT) is the last resort in the treatment of a wide variety of acute and chronic end stage liver diseases (ESLD) in children. A noticeable improvement in survival rates has been achieved with advances in surgery and improved immunosuppressive protocols. However, infections acquired post-LT are still common and can be associated with high morbidity and mortality [1,2,3]. Although management of infections post-transplant has progressed over the past two decades, infections, especially of viral etiologies, still have an impact on the graft function and overall outcome. Hence, a coordinated approach is warranted to mitigate the risks related to infections and graft [4].

The pediatric population is not young adults. They suffer from a wide spectrum of diseases that can lead to either acute or chronic ESLD. The most common ESLD leading to liver transplant is biliary atresia (50%), while the remainder is a combination of inherited metabolic conditions, such as progressive familial intrahepatic cholestasis syndromes. The complications of ESLD that necessitate liver transplant are synthetic failure (prolonged prothrombin time, low albumin), encephalopathy, and failure to thrive. Intractable itching causing poor quality of life can also be an indication of LT. Children awaiting LT are at increased risk of infections [5]. Moreover, the pediatric population comprises different age groups (neonate, infant, toddler, preschool, school, and adolescents) with different predispositions to infections, and, accordingly, management could vary and pose unique challenges. The patient’s age at the time of transplant determines the possibility of being exposed to pathogens prior to transplant and the set of immunization the patient has received; both can influence the frequency of infections post-transplant [6,7].

Viral infections post-liver transplant includes cytomegalovirus (CMV), Epstein–Barr virus (EBV), herpes simplex virus (HSV), respiratory viruses, and others. Given the young age of recipients, the naïve status of the recipient’s EBV and CMV and old donor age, with a high probability of positive viral serostatus, accounts for higher rates of post-transplant viral infections. This necessitates close monitoring, identifying risk factors, and preemptive therapy or prophylactic treatment, depending on the center policy. The effects of viral infections can be direct or direct. Fever, invasive infections such as pneumonia, enteritis, meningitis, and encephalitis, or neutropenia are direct effects. The indirect effect is immune system alteration due to the release of chemokines and growth factors secondary to viral infections. Some viral infections can trigger graft rejection, which may be due to changes in the expression of surface antigens, such as histocompatibility antigens.

The aim of our study was to review the viral infections’ epidemiology and describe risk factors and patient outcomes after LT.

## 2. Materials and Methods

The medical records of ninety-six children who underwent liver transplant between 2017–2018 at the Pediatric Liver Centre, at Kings College Hospital, were reviewed retrospectively. Demographic, clinical, and laboratory data for all liver transplant recipients (LTR) were collected. The LTR data included the patient age, underlying acute or chronic illness, and bacterial or viral infections before and after transplant. Donor data included donor age, CMV status, graft type—either whole, right or left lobe, or left lateral segment—deceased or living donor, and donation after circulatory death (DCD) or after brain death (DBD).

Viral infections encountered included CMV infection, EBV infection, and non-EBV non-CMV viral infections including herpes simplex virus (HSV), norovirus, parvovirus, and respiratory viruses such as adenovirus, influenza, parainfluenza, rhinovirus, metapneumovirus, and enterovirus. The common upper respiratory tract viruses were detected using a Bio Fire Film Array respiratory panel or by in-house multiplex polymerase chain reaction (PCR) from nasopharyngeal swabs.

All LTR routinely had weekly CMV and EBV DNA monitoring till discharge from hospital, and on regular follow ups. The EBV and CMV viral DNA was detected in EDTA blood using quantitative multiplex PCR assay (detection limit for both CMV and EBV is <10 copies/mL). The institute antiviral and immunosuppression protocols were followed. Antiviral prophylaxis was initiated for LTR at high risk for CMV infection, i.e., donor CMV IgG positive and recipient CMV IgG negative (D+R−) with intravenous (IV) ganciclovir 5 mg/kg once daily for 2 weeks. Treatment of CMV infection was with IV ganciclovir 5 mg/kg twice daily till two consecutive CMV DNA a week apart were negative. No antiviral was used for EBV viraemia. The reduction in immunosuppression was considered in patients with EBV viremia. The institute immunosuppression protocol was methyl prednisolone in a dose of 2 mg/kg/day and tacrolimus, which is titrated to maintain a level of 10–15 µg/L for LT and 15–20 µg/L for multivisceral transplant. Azathioprine, mycophenolate mofetil, and sirolimus were individualized. Rituximab is considered in cases of rising EBV viraemia where reduction in immunosuppression is not possible due to associated graft dysfunction secondary to allograft rejection.

Risk factors for EBV, CMV, and non-EBV, non-CMV viral infections were analyzed (Section 3.2). CMV and EBV infection and disease definitions were adapted from the American society of transplantation infectious diseases guidelines [8].

### Statistical Analysis

Descriptive statistics on baseline variables were reported as frequency (percentage) for categorical variables and as quartiles (median, 25th and 75th percentiles) for continuous variables. Student’s t test and the Mann–Whitney test were used to analyze continuous variables, and the Chi square test (Fisher exact used if cells < 5) was used to analyze categorical variables. Variables with *p* < 0.05 in the univariate analysis were included in the multivariate analysis. Multivariate analysis used multinominal logistic regression tests. Statistical analysis was performed using SPSS version 20 for Windows (IBM Co., Armonk, NY, USA). Statistical significance was considered achieved at *p* values less than 0.05.

## 3. Results

### 3.1. Demographic and Clinical Characteristics

Over a 2-year duration, 96 pediatric patients underwent transplantation. Isolated LT was performed in 90 (93.8%) patients, multivisceral in 5 (5.3%) patients, and combined liver and kidney transplantation in 1 patient. The median (IQR) age at transplantation was 3 (1–8.75) years. Acute liver failure (ALF) was the indication for LT in 18.8% of patients, and the rest of patients were transplanted for chronic liver disease. Biliary atresia was the most common underlying diagnosis in >50% of patients with chronic liver disease. Forty-two (43.8%) patients had a split graft. Tacrolimus and steroids were administered in all patients, while mycophenolate mofetil (MMF), azathioprine, and sirolimus were used as an add-on immunosuppression in 17 (17.7%) patients. Important relevant complications included biliary anastomotic, bowel perforation, and re-transplantation, which were encountered in 14 (14.6%), 5 (5.2%), and 5 (5.2%) patients, respectively.

### 3.2. Characteristics of Viral Infections after Transplant

Overall, viral infection was found in 73 (76%) patients. The incidence of EBV viremia was 60.4%, CMV infection 35.4%, and of non-EBV non-CMV viruses 30.2%, predominantly adenovirus and rhinovirus.

#### 3.2.1. EBV Infections

Older donor age, auxiliary graft, and bacterial infections were statistically significant risk factors for EBV infection (*p*-value 0.009, 0.025, 0.003) (Table 1). Twelve patients received rituximab; 10 patients had primary EBV infection within the 1st month after transplant and 2 patients received it for B-cell post-transplant lymphoproliferative disease (PTLD).

A logistic regression was carried out to assess the effect of significant factors from univariate analysis (donor age, auxiliary transplant, bacterial infection, and DCD) on the likelihood of having EBV viremia.

The overall model was statistically significant when compared to the null model, χ^2^(4) = 16.7, *p* = 0.002. Bacterial infection (*p* = 0.031) was significant, but DCD (*p* = 0.25), auxiliary transplant (*p* = 0.3) and donor age (*p* = 0.18) were not significant. Bacterial infection was associated with an increased likelihood of EBV viremia.

#### 3.2.2. CMV Infection

Median (IQR) timing for developing CMV infection was 42 (30.75–66) days. Younger recipient age, D+R− CMV IgG, and left lateral segment graft were significant risk factors for CMV infection (*p*-value 0.021, 0.000, 0.005). On the contrary, D-R− CMV IgG and non-split grafts had less risk of CMV infection (*p*-value 0.000, 0.024) (Table 2).

A logistic regression was carried out to assess the effect of patient’s age, D+/R− CMV status, D−/R− CMV status, left lateral segment, non-split graft, and use of azathioprine in immunosuppression on the likelihood of having CMV viremia. The overall model was statistically significant when compared to the null model, χ2(6) = 33.76, *p* = 0.000. D+/R− CMV status (*p* = 0.013) and D−/R− CMV status (*p* = 0.04) were significant, but patient’s age (*p* = 0.9), left lateral segment (*p* = 0.36), non-split graft (*p* = 0.73), and use of azathioprine (*p* = 0.135) were not significant. D+/R− CMV status was associated with increased likelihood of CMV viremia, and D−/R− CMV status was associated with a decreased likelihood of CMV viremia.

#### 3.2.3. EBV and CMV Viral Infections

Twenty-five patients had both EBV and CMV infections post-transplant. Their median (IQR) age was 1.2 (1–3) years. Twenty-one patients were transplanted for underlying chronic liver disease and four for acute liver failure. The graft was left lateral segment in 84% of EBV/CMV infected patients. The median (IQR) length of hospital stay was 29 (21–49.5) days and there was 1 mortality among this group of patients.

#### 3.2.4. Non-EBV Non-CMV Viral Infections

Twenty-one (23%) patients had 21 episodes of non-EBV non-CMV viral infections before transplant and 29 (30.2%) patients had 33 episodes of non-EBV non-CMV viral infections after transplant. Adenovirus and rhinoviruses were the predominant infections (Table 3). More than 70% of patients with non-EBV non-CMV viral infections before transplant continued to have infections after transplant. Pretransplant viral infections were the only risk factors for developing non-EBV non-CMV viral infections post-transplant (*p*-value 0.003) (Table 4).

## 4. Discussion

Children are more vulnerable to viral infections, particularly CMV and EBV, given the recipient’s naïve status at time of transplant and the likely positive serology of the donor graft. This poses a risk of acquiring primary infection or reactivation after transplant while under immunosuppression.

Two thirds of our patients had EBV viremia, and most patients with high EBV viremia were subjected to reduction in their immunosuppression. Rituximab was administered in 20% of patients who showed persistently rising EBV viremia in the first month after transplant and where immunosuppression could not be reduced. In our univariate analysis, older donor age carried a risk of developing EBV viremia. Exposure to EBV increases with age, with subsequent positive serology facilitating EBV transmission from older donor grafts to naïve immunocompromised pediatric recipients. Auxiliary graft is unique to our center, and a significant high proportion of patients who had auxiliary liver transplant had EBV viremia; however, the cause for this association was unclear. Bacterial infections were associated with increased likelihood of developing EBV viremia on both univariate and multivariate regression analysis. This may be attributed to alteration of the underlying immune system in the setting of immunosuppression after LT.

Incidence of EBV infection in different studies varied according to the aim of the study; it has been reported as 8.3–50% for EBV infection and 2.6% for post-transplant lymphoproliferative disease (PTLD) [9,10]. Risk factors in these studies included EBV negative recipients, younger patient age, frequent rejection episodes, and higher doses of immunosuppression [9,10]. The SPLIT group study showed that frequent rejection episodes were associated with symptomatic EBV and PTLD, while in contrast we observed no significant impact on graft rejection [10]. Weiner et al. (2012) reported that graft rejection was a significant risk factor for developing PTLD and that most patients with PTLD will eventually develop graft rejection [11]. Therefore, close monitoring is necessitated in any case of EBV viremia and immunosuppression reduction for possibility of graft rejection.

The immune system is innately immature in infants, and thus likely to be CMV naïve; however, they are at less risk of rejection. Accordingly, pediatric LTR requires a meticulous age-specific review of the type and dose of immunosuppression, which has to be balanced against the relatively lower rejection risk and higher risk of infection [12]. Younger patients were more likely to develop CMV infections, which is due to less exposure and negative serology. CMV mismatch significantly predicted CMV viremia on both univariate and multivariate regression. Antiviral prophylaxis administered to this cohort only postponed the CMV infection. Therefore, close monitoring of CMV is mandatory once the patient comes off of antivirals, as they may develop CMV infection after stopping antiviral treatment. On the contrary, D−R− CMV IgG was protective against CMV infection. Nevertheless, those patients with D−R− CMV IgG should be considered for CMV negative blood products during the first few months post-transplant, while still on high immunosuppressive doses. Left lateral segment graft was another risk factor on univariate analysis for CMV infection, while recipients of whole non-split organ were at less risk. This may be related to patient age; a young recipient is transplanted with either a left lateral segment from an adult (D+R−), with higher risk of CMV acquisition, or with a whole non-split graft of a child (D−R−). However, the type of graft was not significant in multivariate analysis.

Incidence of CMV infection varied widely in different studies, ranging from 15–70% [13,14]. This variation could be explained due to heterogeneity of protocols in management of CMV surveillance and antiviral prophylaxis. A previous study from our center showed higher percentages of CMV infection (60%) for pediatric LTR on long term follow up, up to 2 years [5]. In contrast, a Turkish study reported low incidence of CMV (23.4%) and EBV (15.6%) infection, likely due to less frequent monitoring of CMV and EBV DNA [15]. It is noticeable that viral infections in adults post-LT does not impose a major burden, as it does in the pediatric population. Some studies have shown that the overall viral infections were 15%, with CMV representing the majority of cases [16]. Similar to other studies, we observed that grafts from CMV antibody-positive donors to CMV antibody-negative recipients remain a significant risk factor for CMV infection [13]. However, we reported an increased incidence of CMV hepatitis in D−R+ [5]. Theoretically, CMV D−R+ grafts will be more prone to infection with CMV viraemia. There is a need for more data to understand the role of antiviral prophylaxis in this group. Unlike a study from a research group in the US, rejection was not a risk factor for CMV infection in our cohort of patients [12].

Non-EBV non-CMV viral infections were as common as CMV infections. However, it was recognized that respiratory viruses (rhinovirus, metapneumovirus, parainfluenza, flu, respiratory syncytial virus) constituted a significant portion of post-transplant non-EBV non-CMV viral infections. A multicenter consortium analyzing the data of pediatric recipients with solid organ transplant who developed respiratory viral infections post-transplant reported that 15% of LTR developed viral respiratory infections, and the younger patients were more prone to pulmonary complications [17]. In our cohort of patients, pre-transplant viral infections posed a significant risk for developing post-transplant infections. Although non-EBV non-CMV viral infections have no impact on morbidity and mortality post-LT, it is debatable if transplant should be delayed till clearance of infection. In our center, we wait approximately 2–3 weeks after the acute respiratory infection for transplant surgery in non-urgent LTR. However, consensus guidelines, based on multi-center surveys, recommended that the decision of transplant surgery should be tailored based on how urgent the transplant is and the type and severity of infecting pathogen [18].

## 5. Conclusions

Pediatric LTR had a high incidence of viral infections. Despite high prevalence of viral infections in our cohort, EBV, CMV and non-EBV non-CMV viral infections were not associated with more rejection or morbidity or mortality. Pre-transplant non-EBV non- CMV viral infections did not contribute to increased complications after transplant. Although some of the risk factors for viral infections are unavoidable, identifying the characteristics and risk pattern will help improve the care for pediatric LTR.

## 6. Limitations of the Study

This is a retrospective study, and the variable underlying diagnosis and age could have impact on viral infections. Some of the donor data, e.g., EBV serology, was not available to identify high risk patients for EBV infection. We cannot ascertain the timing of onset of all viral infections as the onset dates were not always documented. We have included 6 LTR who also had intestinal transplant, and one had renal transplant included in the data analysis; these patients had longer durations of antiviral prophylaxis compared to isolated liver LTR.

## Figures and Tables

**Table 1:** Demographic and risk factors for Epstein–Barr virus (EBV) viremia in pediatric liver transplant recipients.

	No EBV Viremia (N = 38)	EBV Viremia (N = 58)	*p*-Value
Pretransplant data			
Age (years); median (IQR)	2 (0.75–8.25)	3 (1–9)	0.37
ALF; N (%)	6 (15.8)	12 (20.7)	0.55
CLD; N (%)	32 (84.2)	46 (79.3)	0.55
Time from assessment to transplant (days); median (IQR)	90 (12–259.5)	73 (30–210)	0.42
Donor age (months); median (IQR)	19 (11–29)	30 (18.5–39)	0.009 *
DCD	9 (23.7)	3 (5.2)	0.011 *
DBD	23 (60.5)	40 (69)	0.395
Transplant data			
Graft type; N (%)			
LLS	21 (55.3)	37 (63.8)	0.4
Whole	15 (39.5)	13 (22.4)	0.07
Right lobe	1 (2.6)	5 (8.6)	0.4
Left lobe	1 (2.6)	3 (5.2)	1
Auxiliary transplant; N (%)	1 (2.6)	11 (19)	0.025 *
Post-transplant			
CMV infection; N (%)	9 (23.7)	25 (43.1)	0.052
Bacterial infection; N (%)	5 (13.2)	24 (41.4)	0.003 *
Time to first rejection (days); median (IQR)	17 (10–29)	19 (10–40.25)	0.06
Rejection; N (%)	20 (52.6)	41 (70.7)	0.07
>3 rejection episodes; N (%)	5 (13.2)	9 (15.5)	0.75
Biliary complications; N (%)	4 (10.5)	10 (17.2)	0.55
Bowel perforation; N (%)	3 (7.9)	2 (3.4)	0.38
Retransplant; N (%)	4 (10.5)	1 (1.7)	0.08
PICU stay (days); median (IQR)	5 (2–11.5)	3 (2–11)	0.62
Length of hospital stay (days); median (IQR)	22 (16–39.5)	29 (20.78–45.25)	0.21
Immunosuppression medications; N (%)			
Mycophenolate mofetil	6 (15.8)	11 (19)	0.69
Azathioprine	8 (21.1)	9 (15.5)	0.49
Sirolimus	1 (2.6)	6 (10.3)	0.24
Mortality	4 (10.5)	2 (3.4)	0.2

* *p*-value < 0.05 is significant, ALF; acute liver failure, CLD; chronic liver disease, DCD; donation after circulatory death, DBD; donation after brain stem death, LLS; left lateral segment, CMV; cytomegalovirus.

**Table 2:** Demographic and risk factors for cytomegalovirus infection (CMV) in pediatric liver transplant recipients.

	No CMV Viremia (N = 62)	CMV Viremia (N = 34)	*p*-Value
Pretransplant data			
Age (years); median (IQR)	4 (1–11)	1.6 (1–3.25)	0.021 *
Acute liver Failure; N (%)	13 (21)	5 (14.7)	0.452
Chronic liver disease; N (%)	49 (79)	29 (85.3)	0.452
CMV status (Donor/Recipient); N (%)			
D+R+	13 (21.3)	7 (20.6)	0.93
D+R−	8 (12.9)	19 (55.9)	0.000 *
D−R+	12 (19.4)	5 (14.7)	0.57
D−R−	29 (46.8)	3 (8.8)	0.000 *
Pretransplant bacterial infection	6 (9.8)	2 (5.9)	0.7
Pretransplant viral infection	22 (35.5)	7 (20.6)	0.13
Donor age (months); median (IQR)	22.5 (11.75–38.75)	25 (17.5–35.5)	0.493
DCD	10 (16)	2 (5.9)	0.2
DBD	41 (66.1)	22 (64.7)	0.89
Transplant data			
Graft type; N (%)			
Left lateral segment	31 (50)	27 (79.4)	0.005 *
Whole	22 (35.5)	6 (17.6)	0.066
Right lobe	5 (8.1)	1 (2.9)	0.42
Left lobe	4 (6.5)	0	0.294
Partition; N (%)			
Split	26 (41.9)	16 (47.1)	0.63
No split	27 (43.5)	7 (20.6)	0.024 *
Living donor	9 (14.5)	10 (29.4)	0.08
Reduced	-	1 (2.9)	
Auxiliary transplant; N (%)	10 (16.1)	2 (5.9)	0.2
Post-transplant			
EBV viremia; N (%)	33 (53.2)	25 (73.5)	0.052
Bacterial infection; N (%)	17 (27.4)	12 (35.3)	0.42
Time to first rejection (days); median (IQR)	17 (10–35)	24 (10–31)	0.37
Rejection, N (%)	38 (61.3)	23 (67.6)	0.54
>3 rejection episodes, N (%)	8 (12.9)	6 (17.6)	0.53
Patients receiving rituximab; N (%)	6 (9.7)	6 (17.6)	0.259
Biliary complications; N (%)	9 (14.5)	5 (14.7)	0.98
Bowel perforation; N (%)	3 (4.8)	2 (5.9)	1
Retransplant; N (%)	3 (4.8)	2 (5.9)	1
PICU stay (days); median (IQR)	5 (2–11.75)	3 (2–9.25)	0.33
Length of hospital stay (days); median (IQR)	28 (17.5–45.5)	26 (21–37)	0.99
Immunosuppression medications; N (%)			
Mycophenolate mofetil	12 (19.4)	5 (14.7)	0.568
Azathioprine	15 (24.2)	2 (5.9)	0.027 *
Sirolimus	5 (8.1)	2 (5.9)	1
Mortality	4 (6.4)	2 (5.9)	1

* *p*-value < 0.05 is significant, DCD; donation after circulatory death, DBD; donation after brain stem death.

**Table 3:** Frequency and type of pre- and post-transplant non-EBV non-CMV viral infections among 96 transplanted patients.

Viruses	Pre-Transplant (21 Patients/96)	Post-Transplant (29 Patients/96, 33 Episodes)
Adenovirus	7	14
Rhinovirus	4	9
Parvovirus	2	2
Herpes simplex virus	0	2
Meta-pneumovirus	2	2
Human Herpes virus 8	0	1
Para-Influenza 1	1	1
Para-influenza 3	2	1
Respiratory syncytial virus	1	1
Influenza A	1	0
Norovirus	1	0

**Table 4:** Demographic, clinical and laboratory data in transplanted patients who did not have non-EBV non-CMV infections (N = 67) and those who had non-EBV non-CMV infections (N = 29).

	No Non-EBV Non-CMV Infection (N = 67)	Non-EBV Non-CMV Infection (N = 29)	*p*-Value
Pretransplant data			
Age (years); median (IQR)	4 (1–10)	2 (1–3.5)	0.101
Acute liver failure; N (%)	10 (14.9)	8 (27.6)	0.14
Chronic liver disease; N (%)	57 (85)	21 (72.4)	0.14
Pretransplant viral infection	14 (20.9)	15 (51.7)	0.003 *
CMV status (Donor/Recipient); N (%)			
D+R+	14 (20.9)	6 (20.7)	0.95
D+R−	17 (25.4)	10 (34.5)	0.36
D−R+	10 (14.9)	7 (24.1)	0.26
D−R−	26 (38.8)	6 (20.7)	0.08
Donation after circulatory death (DCD)	9(13.4)	3 (10.3)	1
Donation after brain stem death (DBD)	43 (64.2)	20 (69)	0.65
Transplant data			
Graft type; N (%)			
Left lateral segment	40 (59.7)	18 (62.1)	0.83
Whole	21 (31.3)	7 (24.1)	0.48
Right	5 (7.5)	1 (3.4)	0.66
Left	1 (1.5)	3 (10.3)	0.8
Partition; N (%)			
Split	29 (43.3)	13 (44.8)	0.89
No split	23 (34.3)	11 (37.9)	0.73
Living donor	15 (22.4)	4 (13.8)	0.4
Reduced	-	1 (3.4)	
Auxiliary transplant; N (%)	8 (11.9)	4 (13.8)	0.8
Post-transplant			
CMV infection; N (%)	22 (32.8)	12 (41.4)	0.42
EBV viremia; N (%)	38 (56.7)	20 (69)	0.26
Bacterial infection; N (%)	18 (26.9)	11 (37.9)	0.28
Time to first rejection (days); median (IQR)	19.5 (10.25–31.75)	16.5 (8.25–37.75)	0.757
Rejection; N (%)	44 (65.7)	17 (58.6)	0.5
>3 rejection episodes; N (%)	7 (10.4)	7 (24.1)	0.08
Biliary complications; N (%)	9 (13.4)	5 (17.2)	0.63
Bowel perforation; N (%)	4 (6)	1 (3.4)	1
Retransplant; N (%)	4 (6)	1 (3.4)	1
PICU stay (days); median (IQR)	3.5 (2–10.75)	5 (2–11.75)	0.62
Length of hospital stay (days); median (IQR)	27 (20–40)	29 (22–49)	0.43
Immunosuppression medications; N (%)			
Mycophenolate mofetil	9 (13.4)	8 (27.6)	0.095
Azathioprine	16 (23.9)	1 (3.4)	0.01 *
Sirolimus	4 (6)	3 (10.3)	0.4
Mortality	3 (4.5)	3 (10.3)	0.36

* *p*-value < 0.05 is significant, DCD; donation after circulatory death, DBD; donation after brain stem death.

## Data Availability

Not applicable.
